# Impact of quality of evidence on the strength of recommendations: an empirical study

**DOI:** 10.1186/1472-6963-9-120

**Published:** 2009-07-21

**Authors:** Benjamin Djulbegovic, Thomas A Trikalinos, John Roback, Ren Chen, Gordon Guyatt

**Affiliations:** 1 Center for Evidence-based Medicine and Health Outcome Research, Clinical Translational Science Institute, Florida, USA; 2 H. Lee Moffitt Cancer Center & Research Institute University of South Florida, USA; 3 Center for Clinical Evidence Synthesis, Institute for Clinical Research and Health Policy Studies, Tufts Medical Center, USA; 4 Evidence-based Medicine Component, Tufts Clinical Translational Science Institute, USA; 5 Emory School of Medicine, Emory University, Atlanta, USA; 6 Department of Clinical Epidemiology, McMaster University, Canada

## Abstract

**Background:**

Evidence is necessary but not sufficient for decision-making, such as making recommendations by clinical practice guideline panels. However, the fundamental premise of evidence-based medicine (EBM) rests on the assumed link between the quality of evidence and "truth" and/or correctness in making guideline recommendations. If this assumption is accurate, then the quality of evidence ought to play a key role in making guideline recommendations. Surprisingly, and despite the widespread penetration of EBM in health care, there has been no empirical research to date investigating the impact of quality of evidence on the strength of recommendations made by guidelines panels.

**Methods:**

The American Association of Blood Banking (AABB) has recently convened a 12 member panel to develop clinical practice guidelines (CPG) for the use of fresh-frozen plasma (FFP) for 6 different clinical indications. The panel was instructed that 4 factors should play a role in making recommendation: quality of evidence, uncertainty about the balance between desirable (benefits) and undesirable effects (harms), uncertainty or variability in values and preferences, and uncertainty about whether the intervention represents a wise use of resources (costs). Each member of the panel was asked to make his/her final judgments on the strength of recommendation and the overall quality of the body of evidence. "Voting" was anonymous and was based on the use of GRADE (Grading quality of evidence and strength of recommendations) system, which clearly distinguishes between quality of evidence and strength of recommendations.

**Results:**

Despite the fact that many factors play role in formulating CPG recommendations, we show that when the quality of evidence is higher, the probability of making a strong recommendation for or against an intervention dramatically increases. Probability of making strong recommendation was 62% when evidence is "moderate", while it was only 23% and 13% when evidence was "low" or "very low", respectively.

**Conclusion:**

We report the first empirical evaluation of the relationship between quality of evidence pertinent to a clinical question and strength of the corresponding guideline recommendations. Understanding the relationship between quality of evidence and probability of making (strong) recommendation has profound implications for the science of quality measurement in health care.

## Background

Evidence is necessary but not sufficient for decision-making, such as making recommendations by clinical practice guideline (CPG) panels [1]. Indeed, GRADE, a new emerging system for developing CPG recommendations clearly distinguishes between quality of evidence (the extent to which our confidence in an estimate of effect is correct, i.e. represents the "truth") and strength of recommendations (the extent to which confidence in an estimate of the effect is adequate to support recommendations, i.e. belief that adherence to a particular recommendation will do more good than harm)[2]. The GRADE system that has been developed by the pioneers of evidence-based medicine (EBM) represents the major solidification of the entire EBM movement, which, at its center piece, has always focused on the issues of (quality of) evidence and decision-making (guidelines recommendations). In fact, it can be argued that the fundaments of EBM rest on the assumed link between the quality of evidence and "truth" and/or correctness in making recommendations for practitioners and patients alike[3]. If this assumption is an accurate one, then the quality of evidence ought to play a key role in making guidelines recommendations. Surprisingly, and despite the fact that opinion leaders concluded that EBM represents one of the most important medical milestone of the last 160 years, in the same category of innovations such as antibiotics and anesthesia[4], no empirical research to date has investigated the impact of quality of evidence on strength of recommendations made by CPG panels. Here, we report such a study.

## Methods

The American Association of Blood Banking (AABB) has recently convened a panel to develop CPG for the use of fresh-frozen plasma (FFP) for 6 different clinical indications (manuscripts in preparation). The panel decided to employ the GRADE method[2,5] to develop guidelines for 6 clinical indications regarding the use of FFP. The panel was composed of 17 members; 11 were representatives of the AABB Clinical Transfusion Medicine Committee, while 6 were representatives of other professional organizations including the American Association for the Study of Liver Disease (AASLD), the American Academy of Pediatrics (AAP), the American Society of Anesthesiologists (ASA), the American Society for Hematology (ASH), and the military. Nine of the members were pathologists and/or hematologists, two were anesthesiologists, three were internists, two were pediatricians, and one was a hepatologist. The panel was aided by three methodologists: two who performed the systematic review and one who moderated and assisted the panel in their deliberations. None of the methodologists had "voting" power. The panel met in person once during a whole day meeting where the principles of the GRADE methods were introduced and discussed. The panel subsequently had 3 more conference calls to discuss the evidence and formulate the guidelines (see below).

GRADE's fundamental premise is that the CPG should be based on systematic review (SR) of evidence, i.e. a SR of the totality of relevant highest quality research evidence represents a scientific foundation for development of clinical practice guidelines[5]. The AABB commissioned a SR, which also included preparation of the GRADE evidence profiles[5] summarizing the effect of FFP in various clinical scenarios. The SR was prepared based on the key clinical questions submitted by members of the guidelines panel. The evidence profile displayed information on the effect of FFP in terms of benefits and harms for the most important clinical outcomes (e.g. death, transfusion-associated lung injuries etc). The effects were presented both in terms of absolute and relative effect measures. Following the GRADE method, the profiles also contained information on study limitations, (in)consistency,(in)directness, (im)precision and reporting bias as well as the magnitude of FFP treatment effect, whether the confounders were accounted for, and whether there was a dose-response[6] for the use of FFP in given clinical circumstances. These are the factors that GRADE recommends for rating the quality of evidence. For each question/indication, the evidence profiles were given tentative GRADE quality criteria for each outcome of importance by the systematic reviewers (who did not participate in making guideline recommendations). Each member of the panel was sent a full copy of the SR along with the GRADE evidence profile.

According to the GRADE system, **quality of evidence **is rated as:

• **High**: Considerable confidence in the estimate of effect. Future research is unlikely to change the estimate of the health intervention's effect.

•**Moderate**: Further research is likely to have an important impact on confidence in the estimate, and may change the estimate of the health intervention's effect.

•**Low**: Further research is very likely to have an important impact on our confidence in the estimate of effect and is likely to change the estimate.

•**Very low**: Any estimate of effect is very uncertain.

The **strength of recommendations **(for or against intervention) is graded as **strong **(indicating judgment that most well informed people will make the same choice), **weak **(indicating judgment that a majority of well informed people will make the same choice, but a substantial minority will not), or **uncertain **(indicating that the panel made no specific recommendation for or against interventions). The panel was instructed that 4 factors should play a role in making recommendation: quality of evidence, uncertainty about the balance between desirable (benefits) and undesirable effects (harms), uncertainty or variability in values and preferences, and practice setting/uncertainty about whether the intervention represents a wise use of resources (costs)[7]. That is, the panel was given clear explanations and specific situations illustrating how it can, for example, make the strong recommendation even if the quality of evidence is low. Thus, the panel was fully aware that making recommendations depends on the 4 different dimensions and that the quality evidence constitutes only one dimension in formulating the strength of recommendations.

Each member of the panel was asked to make his/her final judgments on the strength of recommendation and the overall quality of the body of evidence. "Voting" was anonymous and were based on the use of GRADE grids for formulation of the strength of recommendations([8]. Each member of the panel had 2 weeks time to formulate his/her judgments on the strength of recommendation and the quality of supporting evidence.

### Statistical analysis

Twelve panel members returned the questionnaire and assessed the quality of evidence and made recommendations for the use of FFP for 6 different clinical indications. We assessed the association between the strength of the recommendation and the corresponding quality ratings as given by each member of the panel. The strength of the recommendations was characterized as "strong" ("for" or "against"), weak ("for" or "against") and "uncertain". Quality (as determined by each panel member) was entered as a categorical outcome ("very low", "low", or "moderate"; since the quality of evidence was never judged to be high- see Results-the outcome "high" was not entered for any panel member for any question). Since "voting" on separate questions by the same member of the panel was likely not independent, we employed a hierarchical, multi-level analysis to analyze the relationship between quality of evidence and the strength of recommendations.

We analyzed the relationship between quality of evidence and the strength of recommendations using a proportional odds model (cumulative logistic model), an extension of logistic regression to handle ordinal response variables. We assessed model fit using a likelihood ratio test (comparing versus an intercept-only model). We asserted that the proportionality of odds assumption was not violated using the score test. The proportional odds model treats all observations as independent, but "votes" on separate questions by the same member of the panel were likely not independent. We explored whether this had any effect by fitting models that allow non-independent observations. Specifically, we fitted a generalized linear mixed model with a cumulative logistic link function and the panel member as the clustering variable, and an additional model using generalized estimating equations. Because the results from the more complicated models were identical with these of the aforementioned simple analysis, we present findings from the ordinal logistic regression (most parsimonious model). Finally, in exploratory analyses we treated the quality ratings given by the panel members as a continuous variable and extrapolated what the predicted probabilities for an uncertain, weak or strong recommendation would be for "high" quality evidence.

All analyses were performed using the *gllamm *program in Stata SE 9.2[9] and using Proc GENMODE in SAS. All p-values are two-tailed and considered significant when less than 0.05.

## Results

Table 1 shows a descriptive analysis portraying a relationship between the quality and strength of recommendations. In our set of data quality ranked from very low to moderate (i.e., was never ranked "high"). The majority of votes clustered in the zone of "very low" evidence and "uncertain" or "weak" recommendations.

**Table 1:** A relationship between the quality (columns) and strength of recommendations (row): a descriptive analysis*

**Strength of Recommendations**	**Quality of Evidence**			**Total**
		
	Very low	Low	Moderate	
**Uncertain**	17	3	0	20

**Weak** **(for or against intervention)**	14	17	4	35

**Strong** **(for or against intervention)**	8	3	6	17

**Total**	39	23	10	72

* the number represent the votes by 12 members of the panel for 6 different clinical indications

In the cumulative logistic model, the odds ratio for stronger recommendations (stronger than "uncertain", as well as stronger than "weak") was 2.06 (95% CI 0.77 to 5.51; p = 0.151) when the quality of evidence was "low" rather than "very low". The corresponding odds ratio was 11.30 (95%CI 2.62 to 48.68; p = 0.001) when the quality of the evidence was "moderate" rather than "very low". Overall, quality was associated with the strength of recommendation beyond chance (p = 0.0027, likelihood ratio test versus an intercept only model). The assumption of proportionality of odds was not violated (Score test: Chi-square = 10.5613, p = 0.1029).

**Figure 1:**
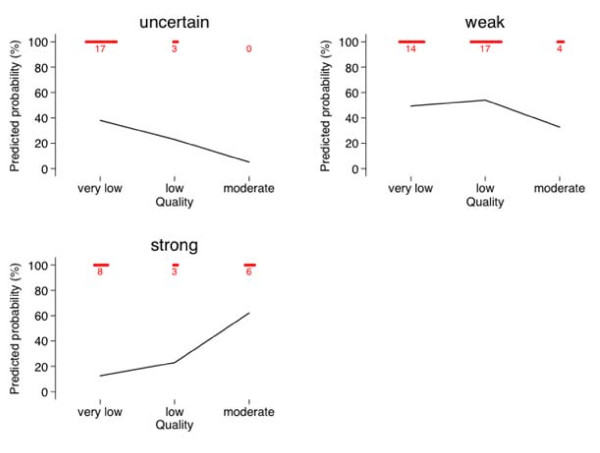
**A relationship between the quality of evidence (X axis) and probability of making uncertain, weak or strong recommendation**. The actual (observed) number of votes is depicted in red in the upper part of the graph.

To facilitate the interpretation of these results, Figure 1 shows the predicted probability (from the cumulative logistic model) for making "uncertain", "weak", or "strong" recommendation (for or against the intervention) as a function of quality. It is evident that the probability for strong recommendation is highest when quality is "moderate" rather than "very low" or "low". Probability of making strong recommendation was 62% when evidence was "moderate", while it was only 23% and 13% when evidence was "low" or "very low", respectively. (Additional file 1 displays distribution of the results for each question).

In exploratory analyses, treating quality as a continuous variable resulted in qualitatively similar estimates. Extrapolating to a hypothetical instance of "high" quality evidence, the predicted probabilities for "strong", "weak" or "uncertain" recommendation was 77.2, 20.2 or 2.6 percent, respectively.

## Discussion

Many factors play a role in making recommendations. As explained above, the GRADE method, which we used to develop the FFP transfusion guidelines, stresses the importance of 4 factors in formulating guideline recommendations: quality of evidence, trade-off between benefits and harms, patient values and preferences, and use of resources.[7] Nevertheless, our analysis indicates that when the quality of evidence increases, the probability of making strong recommendation for or against an intervention dramatically increases. This occurred despite the fact that the panel members were made aware of all factors that should be considered in making recommendations. The quality of evidence had driven the strength of recommendation even in cases of the widespread use of the intervention in medical practice (e.g. FFP is widely used to reverse anticoagulation related to intracranial haemorrhage, yet the majority of the panel members did not strongly endorse it, presumably because of low quality of evidence supporting such a recommendation).

We note with interest that despite the central role that evidence plays in EBM movement, our study is the first empirical study that researched the role of evidence in making guidelines recommendations. The only other study that indirectly investigated a similar issue is one by Cruse et al[10]. These investigators found that when high-quality evidence existed, CPG recommendations were the same regardless which methods were employed in making the recommendations. In other words, strong scientific evidence is capable of serving as a neutral, objective arbiter among competing views and helps generate consensus among parties who, otherwise, may have held the opposite views.[3]

Our study does have some limitations: 1) our study was based on the example from one guideline only (AABB). While we believe that the results from studying other guidelines panels would likely be similar as long these panels use GRADE method, this conjecture remains to be confirmed. 2) the panel members' quality assessment were likely influenced by the quality ratings provided in the SR originally prepared to facilitate developing guidelines recommendations. This quality rating, however, was related to the quality of evidence for each relevant outcome that was analyzed in SR. The panel, on the other hand, rated the quality of the entire body of evidence across all outcomes. Indeed, the fact that the quality rating by 12 members of the panel was not uniform for any of 6 recommendations indicates that each member of the panel was making his/her independent judgments about the overall quality of evidence; 3) we did not collect data on other dimensions of importance for making recommendations such as judgments about trade-offs between benefits and harms, use of resources and patients values and preferences. Although these factors, particularly judgments on trade-off between benefits and harms and use of resources have undoubtedly influenced each member's deliberation, we believe that our results appear to demonstrate that the quality of evidence is likely to be a key factor underlying the strength of the panel's recommendation. 4) due to small sample size (72 observations) and the lack of data on high-quality evidence, we could not establish a more accurate relationship between high quality evidence and strength of recommendations. For 3 categories of data that are related to the quality of evidence (very low, low, moderate) and strength of recommendation (uncertain, weak, strong) our model assumed a linear relationship. A positive relationship exists between the quality of evidence (very low, low, moderate) and strength of recommendation (uncertain, weak, strong). Score test shows the proportional odds assumption that cumulative logit model required is satisfied. However, if we were to have data on the "high" quality category, the relationship may change. In fact, decades of research in psychology and economics show that people's perceptions and judgments are not linearly related. [11]. The same probably applies to making judgments about recommendations: when quality of evidence is low or very low, most people are undecided and are reluctant to endorse a particular intervention. However, when the quality evidence increases, the threshold to make recommendations is crossed, and the probability of making a recommendation likely increases in an exponential fashion. This, however, remains to be demonstrated.

Understanding a relationship between quality of evidence and probability of making (strong) recommendation is important and has profound implications for the science of quality measurements in health care. For example, the GRADE group recommends that adherence to strong recommendations can be used as a quality criterion or performance indicator.[7] We discussed above 4 factors that are important for making guidelines recommendations. All these factors are prone to interpretation and attempting to derive uniformity of judgments about all 4 factors is a very difficult exercise. However, as indicated above, evidence sometimes does succeed in achieving an agreement among rational observers, thus serving as a neutral, objective arbiter among potentially competing views of the panel members. [3] This typically occurs when evidence is uniformly judged to be high. [10] Under these circumstances, our analysis suggests that high quality of evidence would be associated with high probability of making a strong guideline recommendation, which in turn can be the basis for a quality criterion or performance indicator.

## Conclusion

The current work provides an empirical demonstration of the strong link between good quality evidence and decision-making. Because only strong guideline recommendations can be used as performance measures or healthcare quality indicators, it is imperative to design, fund and conduct pragmatic studies that are resistant to internal and external biases. This would ideally generate credible evidence base that will uniformly (close to 100%) be associated with strong recommendations. Thus, efforts to develop performance measures based upon existing research should focus mainly on areas with good quality evidence. Additional research is clearly indicated to establish the true pattern between quality of evidence and strengths of recommendations.

## Competing interests

The authors declare that they have no competing interests.

## Authors' contributions

BD conceptualized the study, performed initial analyses and wrote the first draft of the manuscript. TT and RC performed additional statistical analyses. JR organized AABB guidelines panel and enabled the survey. GG advised on the use and interpretation of the GRADE system. All authors helped interpreted results and edited it for the intellectual content.

## Pre-publication history

The pre-publication history for this paper can be accessed here:



## Supplementary Material

Additional file 1 **Summary of the AABB (American Association of Blood Banking) panel members vote on the use of FFP (fresh-frozen plasma)for 6 different clinical indications**. a distribution of the AABB (American Association of Blood Banking) panel members vote related to the quality of evidence and the strength of recommendations for the use of fresh-frozen plasma for 6 different clinical indications. Click here for file
