# Supplementary material for: Impact of quality of evidence on the strength of recommendations: an empirical study
Source: BMC Health Serv Res. 2009 Jul 21;9:120. doi: 10.1186/1472-6963-9-120 (PMC2722589; doi:10.1186/1472-6963-9-120)
Supplement: Additional file 1 — Summary of the AABB (American Association of Blood Banking) panel members vote on the use of FFP (fresh-frozen plasma)for 6 different clinical indications. a distribution of the AABB (American Association of Blood Banking) panel members vote related to the quality of evidence and the strength of recommendations for the use of fresh-frozen plasma for 6 different clinical indications. [file 1472-6963-9-120-S1.pdf]

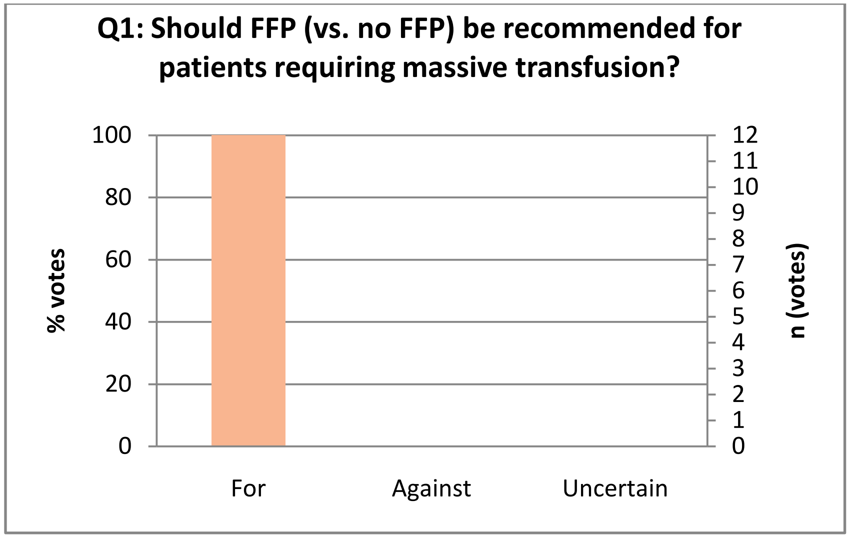
Q1: Should FFP (vs. no FFP) be recommended for patients requiring massive transfusion?
% votes
n (votes)
100
80
60
40
20
0
12
11
10
9
8
7
6
5
4
3
2
1
0
For
Against
Uncertain

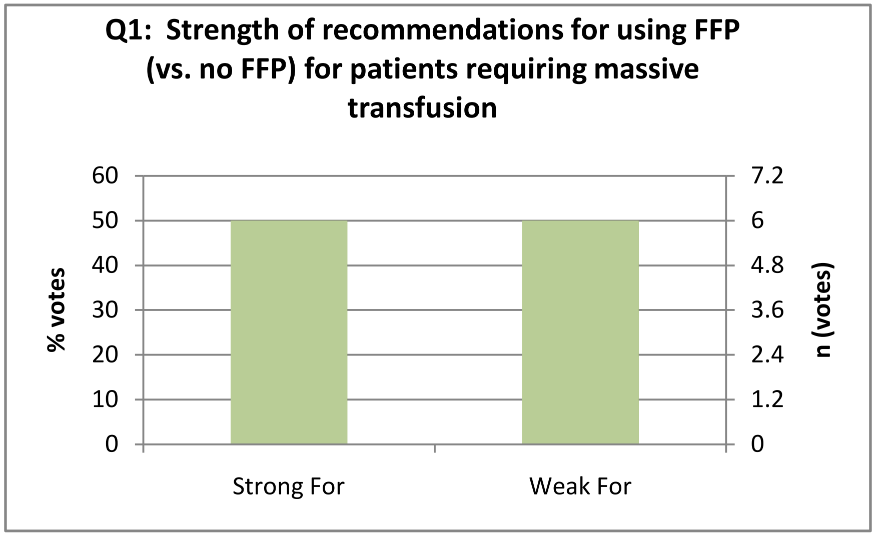
Q1: Strength of recommendations for using FFP (vs. no FFP) for patients requiring massive transfusion
% votes
n (votes)
60
50
40
30
20
10
0
7.2
6
4.8
3.6
2.4
1.2
0
Strong For
Weak For

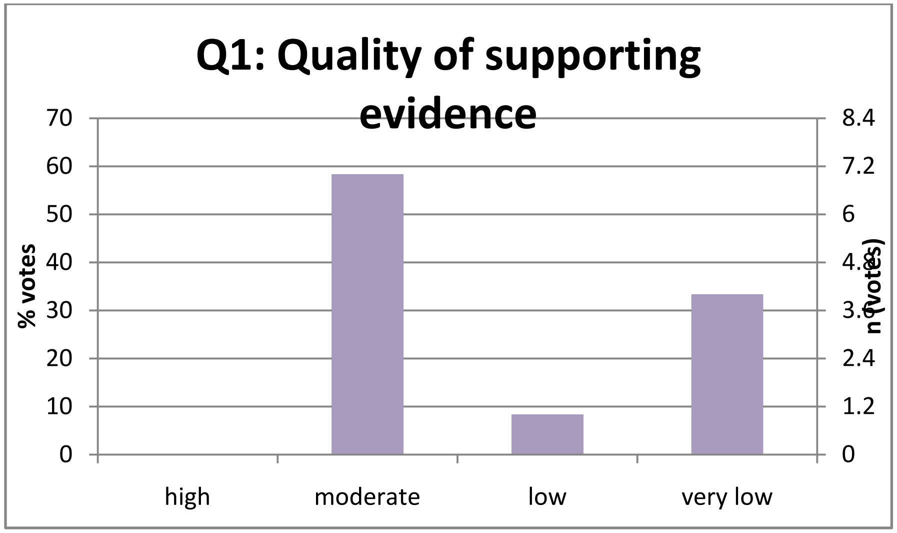
Q1: Quality of supporting evidence
% votes
n (votes)
70
60
50
40
30
20
10
0
8.4
7.2
6
4.8
3.6
2.4
1.2
0
high
moderate
low
very low

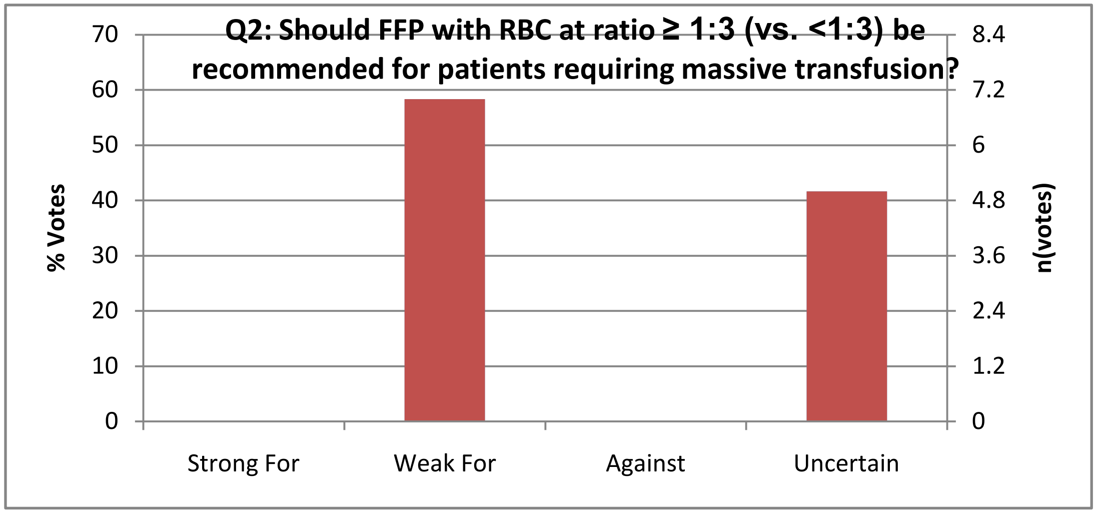
Q2: Should FFP with RBC at ratio ≥ 1:3 (vs. <1:3) be recommended for patients requiring massive transfusion?
% Votes
n(votes)
70
60
50
40
30
20
10
0
8.4
7.2
6
4.8
3.6
2.4
1.2
0
Strong For
Weak For
Against
Uncertain

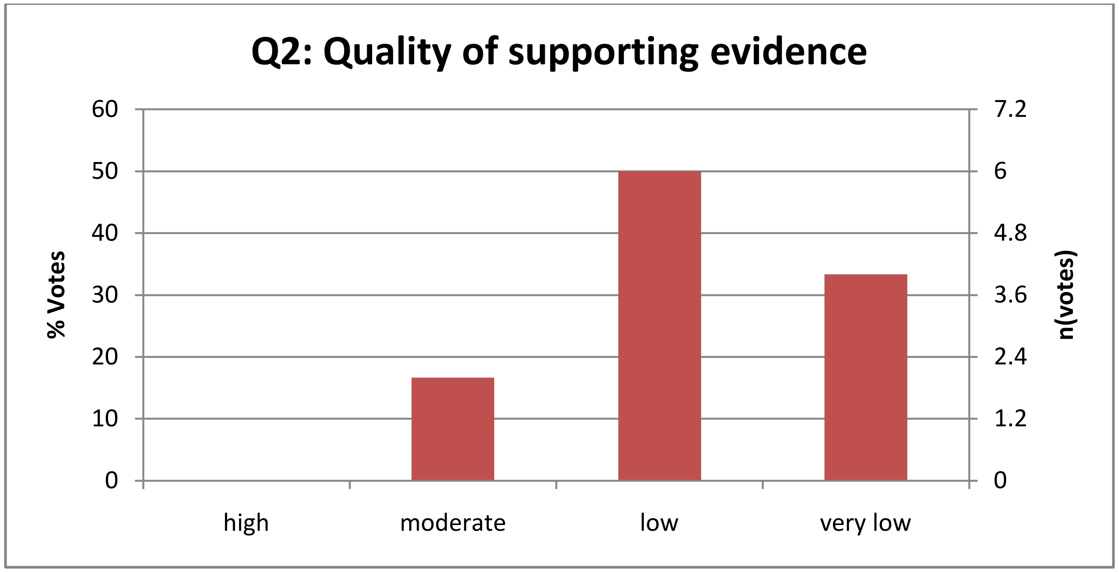
Q2: Quality of supporting evidence
% Votes
n(votes)
60
50
40
30
20
10
0
7.2
6
4.8
3.6
2.4
1.2
0
high
moderate
low
very low

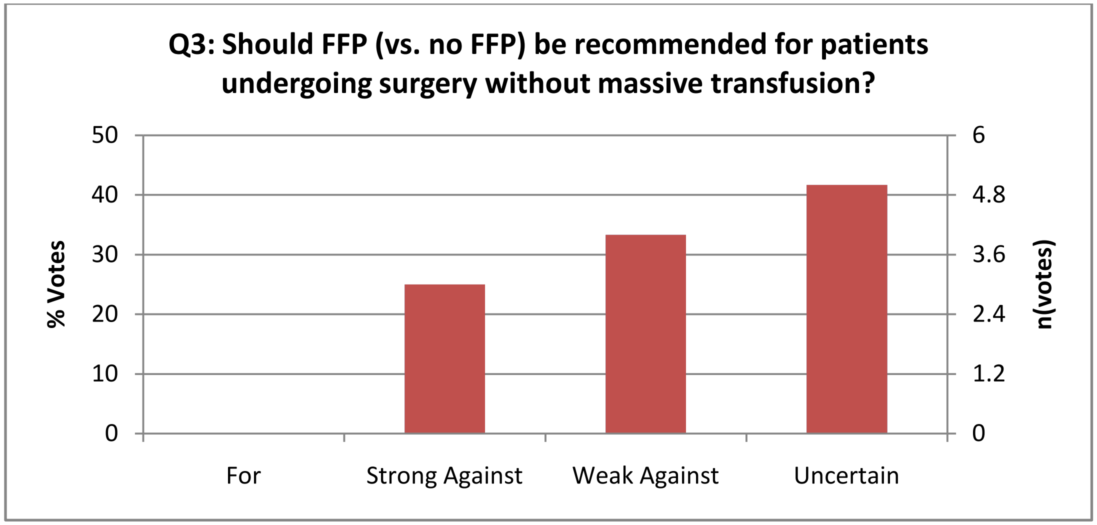

Q3: Should FFP (vs. no FFP) be recommended for patients undergoing surgery without massive transfusion?
50
40
30
20
10
0
% Votes
6
4.8
3.6
2.4
1.2
0
n(votes)
For
Strong Against
Weak Against
Uncertain

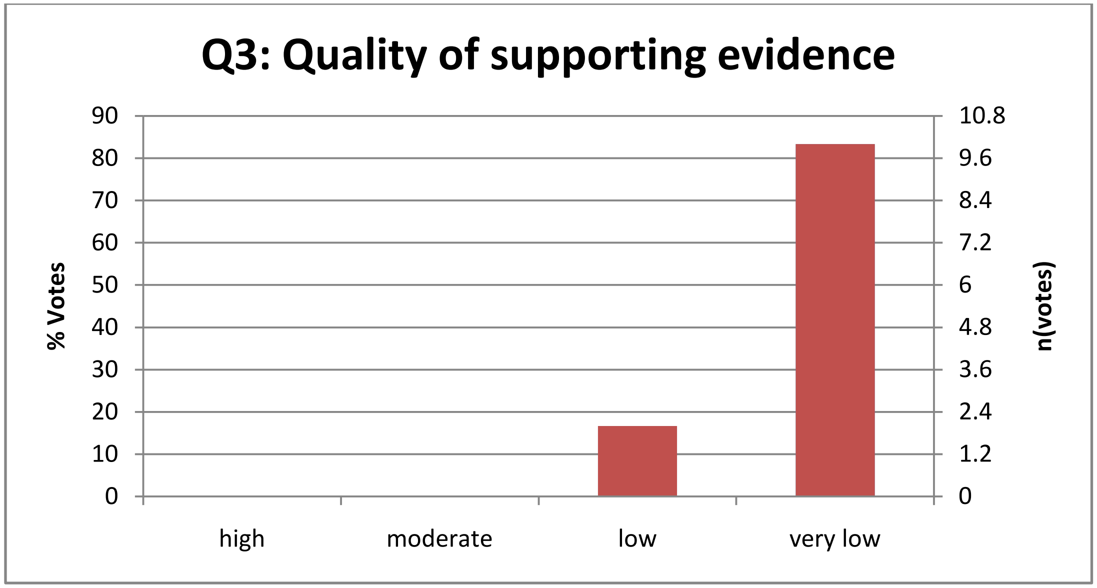

Q3: Quality of supporting evidence
90
80
70
60
50
40
30
20
10
0
% Votes
10.8
9.6
8.4
7.2
6
4.8
3.6
2.4
1.2
0
n(votes)
high
moderate
low
very low

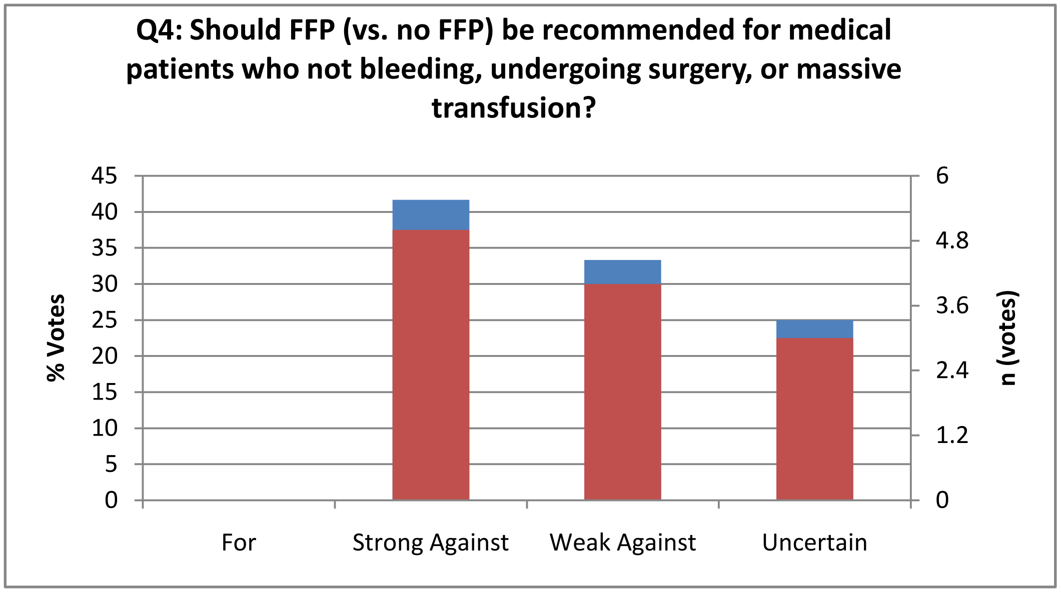
Q4: Should FFP (vs. no FFP) be recommended for medical patients who not bleeding, undergoing surgery, or massive transfusion?
% Votes
n (votes)
45
40
35
30
25
20
15
10
5
0
6
4.8
3.6
2.4
1.2
0
For
Strong Against
Weak Against
Uncertain

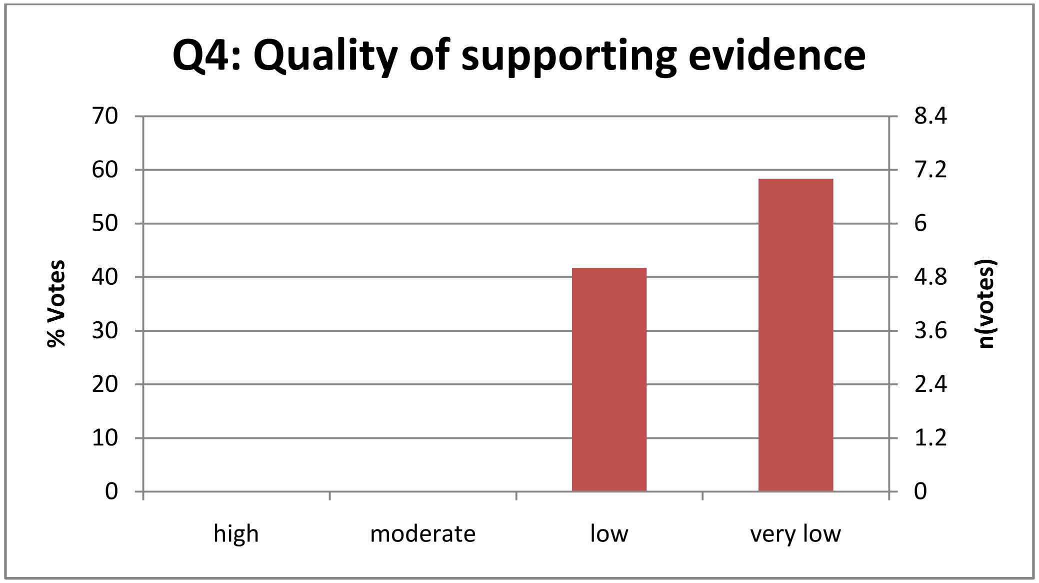
Q4: Quality of supporting evidence
% Votes
n(votes)
70
60
50
40
30
20
10
0
8.4
7.2
6
4.8
3.6
2.4
1.2
0
high
moderate
low
very low

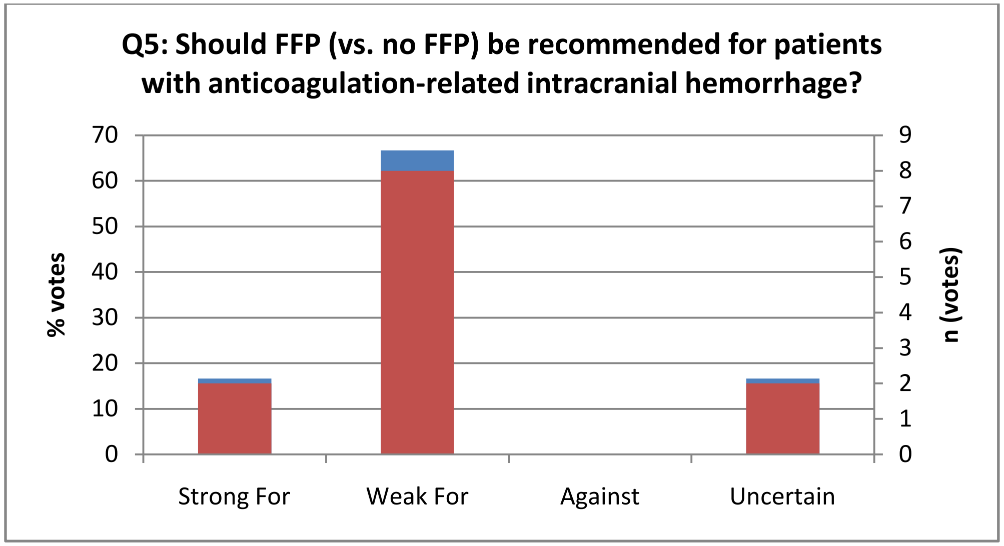
Q5: Should FFP (vs. no FFP) be recommended for patients with anticoagulation-related intracranial hemorrhage?
% votes
n (votes)
70
60
50
40
30
20
10
0
9
8
7
6
5
4
3
2
1
0
Strong For
Weak For
Against
Uncertain

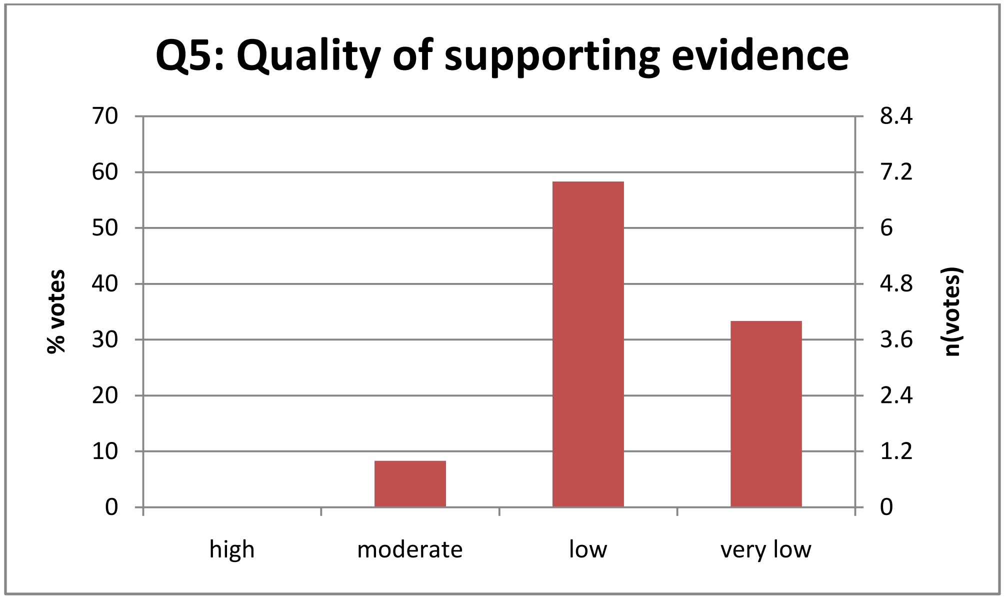
Q5: Quality of supporting evidence
% votes
n(votes)
70
60
50
40
30
20
10
0
8.4
7.2
6
4.8
3.6
2.4
1.2
0
high
moderate
low
very low

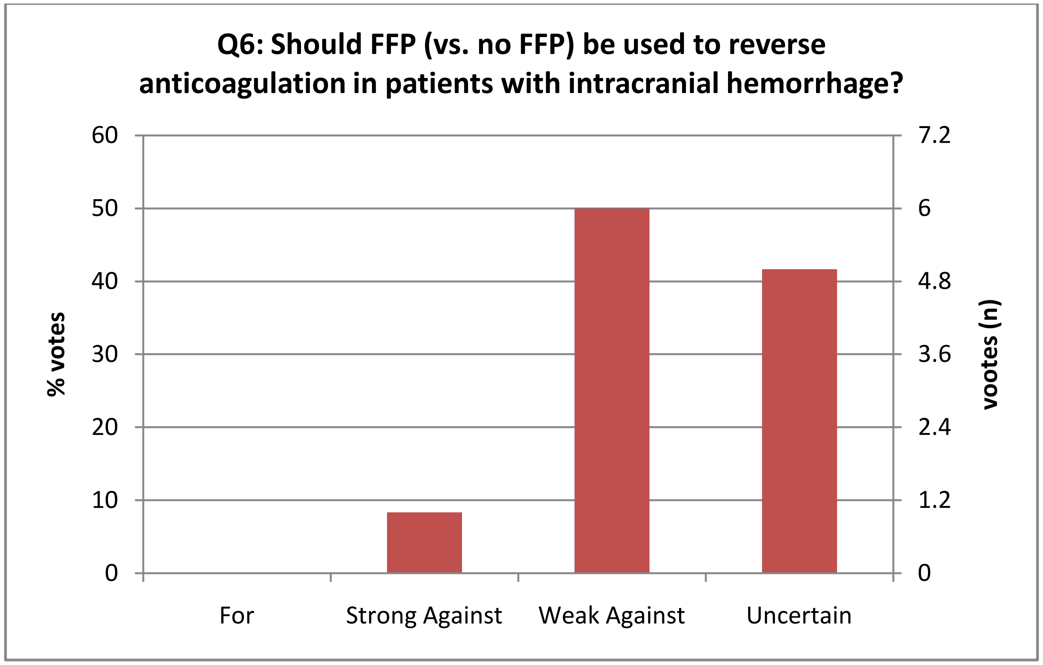
Q6: Should FFP (vs. no FFP) be used to reverse anticoagulation in patients with intracranial hemorrhage?
% votes
vootes (n)
60
50
40
30
20
10
0
7.2
6
4.8
3.6
2.4
1.2
0
For
Strong Against
Weak Against
Uncertain

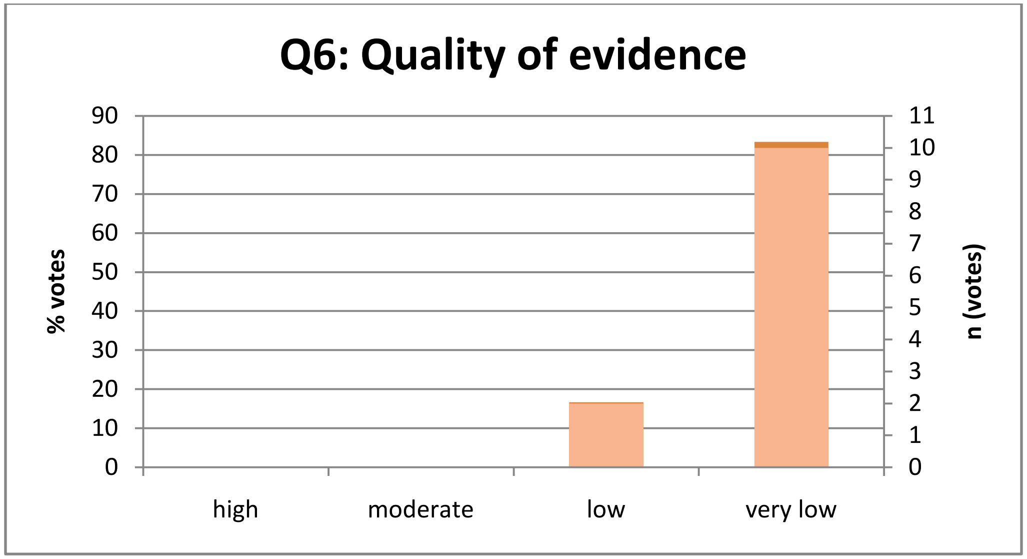
Q6: Quality of evidence
% votes
n (votes)
90
80
70
60
50
40
30
20
10
0
11
10
9
8
7
6
5
4
3
2
1
0
high
moderate
low
very low
